# Carbapenem-resistant hypervirulent *Klebsiella pneumoniae*: molecular evolution, diagnostic markers, and therapeutic challenges

**DOI:** 10.3389/fmicb.2026.1885799

**Published:** 2026-07-03

**Authors:** Tokeshwar Kumar Sahu, Pravin Gadkari, Jaishriram Rathored

**Affiliations:** 1 Department of Microbiology, Jawaharlal Nehru Medical College, Datta Meghe Institute of Higher Education and Research, Wardha, India; 2 Department of Pathology, Jawaharlal Nehru Medical College, Datta Meghe Institute of Higher Education and Research, Wardha, India; 3 Central Research Laboratory and Molecular Diagnostics, Jawaharlal Nehru Medical College, Datta Meghe Institute of Higher Education and Research, Wardha, India; 4 School of Allied Health Sciences, Datta Meghe Institute of Higher Education and Research, Wardha, India

**Keywords:** carbapenemase, carbapenem-resistant *Klebsiella pneumoniae*, CR-hvKp, genomic surveillance, hypervirulent *Klebsiella pneumoniae*, virulence plasmid

## Abstract

Carbapenem-resistant hypervirulent *Klebsiella pneumoniae* (CR-hvKp) is an emerging convergent pathotype in which antimicrobial resistance (AMR) and invasive virulence increasingly overlap. This Mini Review critically summarizes how CR-hvKp arises through acquisition of carbapenemase-encoding plasmids by hypervirulent backgrounds, acquisition of virulence plasmids by established carbapenem-resistant lineages, and formation of hybrid resistance–virulence elements. It also discusses why laboratory recognition remains challenging, since carbapenem resistance, hypermucoviscosity, capsule type, and individual virulence markers are each informative but insufficient alone. A tiered diagnostic approach that integrates susceptibility testing, carbapenemase classification, virulence-marker profiling, capsule and sequence typing, and genome sequencing is therefore more appropriate for high-risk isolates. Therapeutic management remains difficult because available agents must be selected according to carbapenemase class, infection site, source control, susceptibility profile, and risk of resistance emergence. Available evidence supports prioritizing earlier recognition, plasmid-aware surveillance, antimicrobial stewardship, and outcome-focused studies as priorities for improving management of this high-risk convergent pathotype.

## 1 Introduction

*Klebsiella pneumoniae (K. pneumoniae)* exemplifies the convergence of antimicrobial resistance (AMR), bacterial adaptation, and invasive disease. Global AMR estimates show that resistant bacterial infections impose a substantial mortality burden, with Gram-negative organisms contributing importantly across age groups and regions (Naghavi et al., 2024). Within carbapenem-resistant Enterobacterales (CRE), carbapenem-resistant *K. pneumoniae* (CRKP) remains a high-priority organism because of its clinical impact, limited therapeutic options, and capacity for international dissemination (Sati et al., 2025).

The species is clinically heterogeneous, but two pathotypes drive most current concern: classical multidrug-resistant *K. pneumoniae* and hypervirulent *K. pneumoniae* (hvKp). hvKp is clinically distinguished by invasive disease and metastatic complications, particularly pyogenic liver abscess with endophthalmitis, meningitis, or other distant foci, sometimes in patients without major immunosuppression (Russo and Marr, 2019; Choby et al., 2020). Diabetes, immunosuppression, prior antimicrobial exposure, intensive-care stay, invasive devices, and healthcare-associated acquisition may further modify the risk of severe infection, delayed recovery, and healthcare burden (Russo and Marr, 2019; Choby et al., 2020; Tang et al., 2025). Recent cohort evidence indicates that genomically defined hvKp is associated with more severe disease and early mortality, while carbapenem resistance adds therapeutic risk by limiting active treatment options (Tamma et al., 2024; Tang et al., 2025). However, outcome comparisons between CR-hvKp and classical CRKP remain uncertain. A recent systematic review and meta-analysis reported a non-significant trend toward higher mortality in CR-hvKp than in classical CRKP, with estimates strongly influenced by the definition used for hypervirulence (Chen et al., 2025). This uncertainty supports a cautious clinical approach in which CR-hvKp is treated as a high-risk clinical-microbiological phenotype that warrants early assessment for metastatic foci, timely source control when feasible, and close monitoring for treatment failure or relapse.

Carbapenem-resistant hypervirulent *K. pneumoniae* (CR-hvKp) should therefore be viewed as a convergent pathotype rather than as a resistant strain with incidental virulence markers. In this review, resistance–virulence convergence refers to the acquisition, coexistence, or recombination of antimicrobial-resistance determinants and hypervirulence-associated loci within the same clinical isolate, plasmid background, or clonal lineage (Yang et al., 2021; Zhang et al., 2023). Recent genomic evidence suggests that mobilizable plasmids can carry a large share of acquired virulence genes in *K. pneumoniae*, which complicates surveillance because clinically important virulence cargo may move through routes that are less obvious than classical self-transmissible plasmids (Zhang et al., 2023). A 2025 global genomic analysis further identified an IncFIIK34 KPC-2 plasmid as a plausible driver of international CR-hvKp dissemination, reinforcing the need to integrate resistance and virulence detection rather than tracking carbapenemases alone (Jiang et al., 2025b).

Despite this progress, laboratory recognition remains uneven because hypermucoviscosity, capsule type, and virulence-gene carriage do not always map cleanly onto the clinical hypervirulent phenotype (Stanton and Wyres, 2024). Treatment is equally difficult, since current CRE management depends on rapid carbapenemase classification, drug availability, infection site, and patient severity rather than on virulence status alone (Tamma et al., 2024). This Mini Review therefore focuses on the molecular evolution, diagnostic recognition, and therapeutic challenges of CR-hvKp, with emphasis on how convergence biology can be translated into practical microbiology and clinical decision-making.

## 2 Molecular evolution of carbapenem-resistant hypervirulent *K. pneumoniae*

**FIGURE 1:**
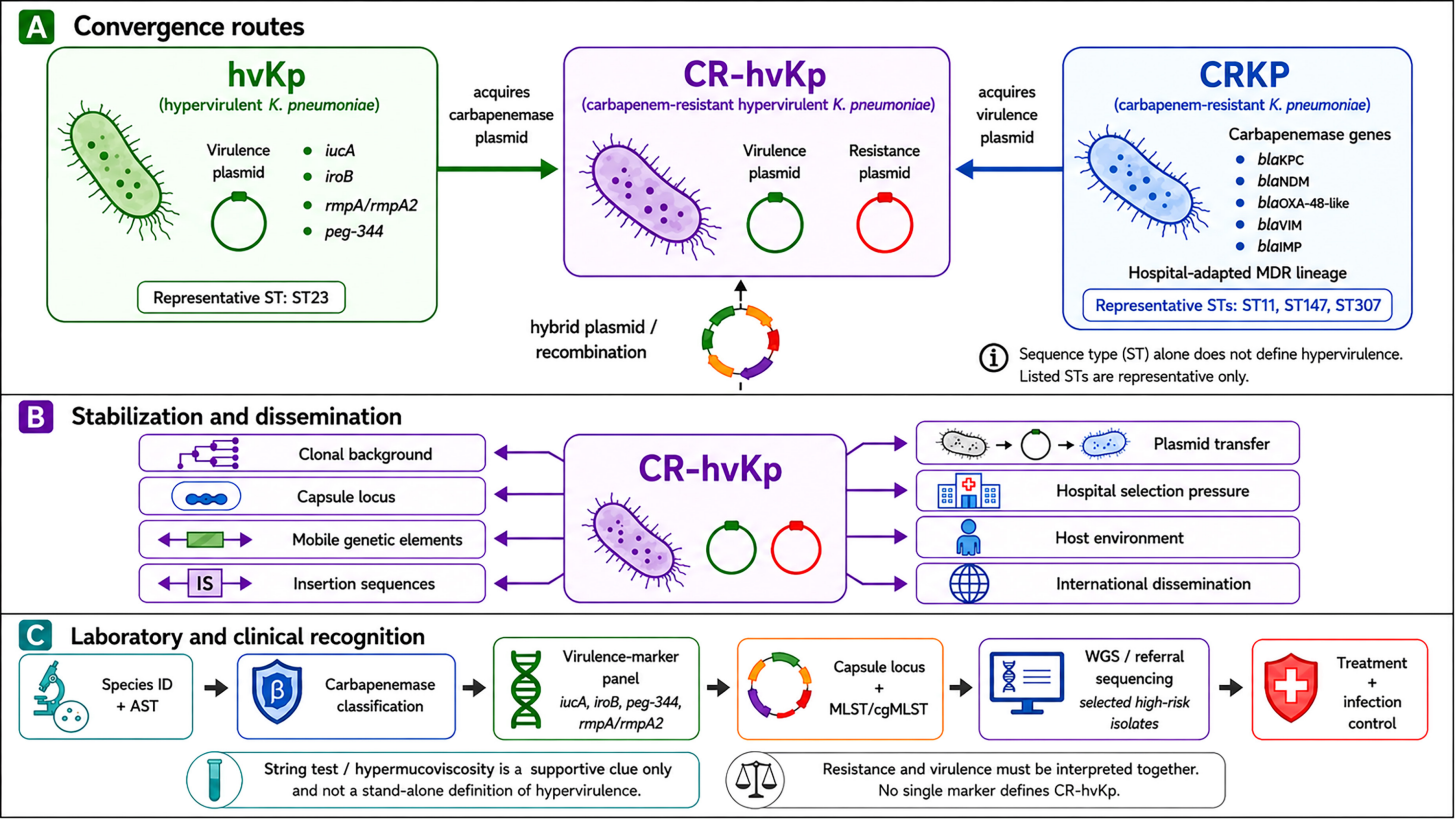
Evolutionary convergence and laboratory recognition of carbapenem-resistant hypervirulent Klebsiella pneumoniae (CR-hvKp). **(A)** Convergence routes leading to CR-hvKp formation. **(B)** Factors involved in stabilization and dissemination. **(C)** Tiered laboratory and clinical recognition workflow. AST, antimicrobial susceptibility testing; ST, sequence type; MLST, multilocus sequence typing; cgMLST, core-genome multilocus sequence typing; WGS, whole-genome sequencing; CR-hvKp, carbapenem-resistant hypervirulent Klebsiella pneumoniae.

Carbapenem-resistant hypervirulent *Klebsiella pneumoniae* has emerged through recurrent convergence rather than a single clonal event. Published genomic and plasmid studies support three principal routes: acquisition of carbapenemase-encoding plasmids by hypervirulent *K. pneumoniae* (hvKp) backgrounds, acquisition of virulence plasmids or virulence loci by established CRKP lineages, and formation of hybrid resistance–virulence plasmids or plasmid-derived elements (Tang et al., 2020; Yang et al., 2021; Han et al., 2022). However, much of the available CR-hvKp literature remains enriched for genomic surveillance studies, outbreak reports, severe case series, experimental phenotyping, and region-specific datasets rather than prospective outcome-linked clinical cohorts; therefore, publication bias, inconsistent hypervirulence definitions, and limited outcome linkage should be considered when interpreting associations between resistance–virulence markers, lineages, and clinical severity (Kochan et al., 2023; Stanton and Wyres, 2024). This distinction is important because plasmid context, gene integrity and expression, capsule background, host factors, and infection site can modulate the observed phenotype (Kochan et al., 2023; Stanton and Wyres, 2024). Figure 1 summarizes the principal evolutionary routes through which resistance and virulence determinants converge in CR-hvKp and links these routes to laboratory recognition.

The first route is exemplified by hvKp strains that acquire plasmids carrying carbapenemase genes such as *bla*KPC, *bla*NDM, or *bla*OXA-48-like determinants. In Singapore, a hypervirulent strain acquired a *bla*KPC-2-bearing plasmid, illustrating how a virulent background can become carbapenem resistant without losing its invasive potential (Chen et al., 2020). The second route is increasingly important in hospital settings, where epidemic CRKP clones acquire virulence plasmids or virulence loci and then expand under antibiotic selection (Zhou et al., 2020; Jiang et al., 2025b). In China, ST11 CRKP has repeatedly served as a genetic platform for this process, especially among KPC-producing lineages with KL64 or related capsule backgrounds (Zhang et al., 2020; Zhou et al., 2020).

Plasmids remain central to this evolution, but the plasmid biology is more complex than simple acquisition of a single virulence plasmid. Conjugative plasmids can disseminate carbapenemase genes, while mobilizable plasmids can move with helper conjugative plasmids and carry a large proportion of acquired virulence genes in *K. pneumoniae* (Zhang et al., 2023; Di Pilato et al., 2024). Zhang et al. (2023) showed that mobilizable plasmids carried more than 75% of acquired virulence genes in their analyzed *K. pneumoniae* dataset, helping explain how virulence traits may spread even when the virulence plasmid itself is not fully self-transmissible. Virulence determinants may also become chromosomally integrated, as shown for an IS26-flanked fragment containing *rmpA2* and *iucABCD-iutA*, creating a potential route for stabilization and subsequent reshuffling of virulence cargo (Yang et al., 2020).

Recent population-level genomics suggests that CR-hvKp dissemination is now being shaped by successful plasmid-lineage combinations. A 2025 global analysis identified an IncFIIK34 KPC-2 plasmid among canonical CR-hvKp strains from 24 countries and reported higher conjugation frequency than the epidemic IncFIIK2 KPC-2 plasmid (Jiang et al., 2025b). These findings shift the evolutionary focus from single markers toward interacting units: clone, capsule locus, carbapenemase plasmid, virulence plasmid, insertion sequence, and host environment.

Within-host evolution adds another layer to this process. During therapy or persistence at specific anatomical sites, CR-hvKp can accumulate mutations that alter resistance, capsule expression, mucoviscosity, or fitness (Jin et al., 2021; Song et al., 2024). This plasticity means that molecular evolution should be viewed as a dynamic process operating across hospitals, plasmid networks, and individual patients.

## 3 Diagnostic markers and laboratory recognition

Laboratory recognition of carbapenem-resistant hypervirulent *K. pneumoniae* should begin from a simple premise: neither carbapenem resistance nor a mucoid colony phenotype is sufficient to define the convergent pathotype. The traditional string test remains useful as a low-cost bedside-to-bench screening tool, but hypermucoviscosity lacks adequate sensitivity and specificity because some classical strains are string-test positive and some genetically virulent strains are string-test negative (Russo and Marr, 2019; Mai et al., 2023; Wahl et al., 2024). This limitation is especially relevant for CR-hvKp, since convergent isolates may carry virulence loci but show variable capsule production, mucoviscosity, serum resistance, neutrophil resistance, and *in vivo* pathogenicity (Kochan et al., 2023; Jiang et al., 2025b).

A practical diagnostic approach should therefore combine phenotypic screening with targeted molecular detection (Table 1). The most widely used genomic markers for predicting hvKp-associated virulence potential are *iucA*, *iroB*, *peg-344*, *rmpA*, and *rmpA2* (Russo et al., 2024; Tang et al., 2025). In strains with acquired AMR, Russo et al. (2024) found that positivity for all five markers provided the highest diagnostic accuracy, whereas positivity for four or more markers maximized sensitivity. A large longitudinal cohort similarly supported a five-marker genomic definition for hvKp prediction, but also showed that genomic classification should not be treated as a complete substitute for clinical and phenotypic assessment (Tang et al., 2025). For CR-hvKp recognition, the main risk is bidirectional misclassification: marker-positive isolates may not express a hypervirulent phenotype if virulence genes are disrupted, weakly expressed, or placed in an unfavorable capsule or plasmid background, whereas marker-incomplete isolates may still warrant concern when invasive disease, metastatic spread, high-risk lineage background, or local epidemiology supports hvKp-like behavior (Kochan et al., 2023; Stanton and Wyres, 2024; Tang et al., 2025). The five-marker panel should therefore be reported as evidence of hypervirulence-associated potential, not as a stand-alone diagnosis of clinical hypervirulence.

**TABLE 1:** Tiered laboratory interpretation and practical feasibility of markers used to recognize carbapenem-resistant hypervirulent *Klebsiella pneumoniae* (CR-hvKp).

Citation	Diagnostic tier	Marker or method	Main contribution	Laboratory complexity and feasibility	Interpretation and key limitation
Tamma et al., 2024	Entry-point resistance screening	Species identification and carbapenem antimicrobial susceptibility testing (AST)	Identifies *K. pneumoniae* with carbapenem nonsusceptibility	Low complexity: feasible in routine microbiology laboratories	Starting point only: carbapenem nonsusceptibility does not define carbapenemase class or hypervirulence
Tamma et al., 2024	Carbapenemase classification	Detection of *bla*KPC, *bla*NDM, *bla*OXA-48-like, *bla*VIM, and *bla*IMP	Links resistance mechanism with treatment selection	Moderate complexity: feasible where phenotypic carbapenemase assays, immunochromatographic tests, or molecular testing are available	Report whenever possible; non-carbapenemase mechanisms and co-produced β-lactamases may complicate interpretation
Russo and Marr, 2019; Russo et al., 2024; Wahl et al., 2024	Hypermucoviscosity screening	String test or quantitative mucoviscosity assay	Provides a rapid phenotypic clue for mucoid behavior	Low complexity for string test	Supportive screen only: hypermucoviscosity and hypervirulence are not equivalent
Russo et al., 2024; Tang et al., 2025	Core virulence-marker panel	*Detection of iucA, iroB, peg-344, rmpA, and rmpA2*	Screens for hvKp-associated virulence potential	Moderate complexity; requires PCR, multiplex testing, or sequencing	Combined marker positivity is stronger than any single marker, but false-positive and false-negative classification remains possible
Wyres et al., 2020; Jiang et al., 2025b	Lineage and capsule context	MLST or cgMLST with capsule-locus typing	Adds epidemiologic, lineage, capsule, and outbreak context	Moderate to high complexity: most useful for surveillance or reference laboratories	Useful for tracking high-risk clone–capsule combinations; K1/K2 status or sequence type alone should not define CR-hvKp
Zhang et al., 2020; Jiang et al., 2025b	Genomic confirmation	Whole-genome sequencing with resistance, virulence, plasmid, and relatedness analysis	Integrates resistance, virulence, clone, capsule, plasmid structure, and transmission	High complexity: best suited to reference laboratories or outbreak investigations	Prioritize for invasive disease, treatment failure, unusual resistance–virulence combinations, or suspected outbreaks; cost, turnaround time, bioinformatics capacity, and plasmid reconstruction remain barriers

AST, antimicrobial susceptibility testing; cgMLST, core-genome multilocus sequence typing; CRE, carbapenem-resistant Enterobacterales; CR-hvKp, carbapenem-resistant hypervirulent *Klebsiella pneumoniae*; CRKP, carbapenem-resistant *Klebsiella pneumoniae*; hvKp, hypervirulent *Klebsiella pneumoniae*; MLST, multilocus sequence typing; WGS, whole-genome sequencing. This table is a narrative synthesis of published studies cited in the table and main text; no unpublished patient-level, laboratory, or experimental data are presented.

For CR-hvKp, the virulence panel must be interpreted together with resistance testing. Carbapenem nonsusceptibility should trigger confirmatory work-up for carbapenemase genes such as *bla*KPC, *bla*NDM, *bla*OXA-48-like, *bla*VIM, and *bla*IMP, because treatment options differ by carbapenemase class (Tamma et al., 2024). A laboratory report that states only “carbapenem resistant” is therefore less clinically useful than one that identifies both the carbapenemase mechanism and the presence or absence of major virulence markers (Tamma et al., 2024; Wahl et al., 2024).

Capsule and sequence typing are best used for epidemiologic context rather than case definition. Although classical hvKp is often associated with K1/K2 backgrounds, CR-hvKp has been reported across broader sequence-type and capsule combinations, including ST11, ST23, ST893, ST147, and ST307 backgrounds (Kochan et al., 2023; Jiang et al., 2025b). Whole-genome sequencing (WGS) provides the most complete integration of lineage, capsule locus, resistance genes, virulence markers, plasmid content, and outbreak relatedness, but cost, turnaround time, and bioinformatics capacity limit routine use (Kochan et al., 2023; Jiang et al., 2025b).

In resource-limited settings, the diagnostic objective should be risk stratification rather than immediate genomic confirmation. A minimum feasible workflow should include species identification, antimicrobial susceptibility testing, documentation of carbapenem nonsusceptibility, and clinical flagging of isolates from invasive syndromes, metastatic infection, treatment failure, or suspected clusters (Tamma et al., 2024). Where available, carbapenemase testing for *bla*KPC, *bla*NDM, *bla*OXA-48-like, *bla*VIM, and *bla*IMP should be prioritized because it directly informs treatment selection (Tamma et al., 2024). The string test may be retained as a low-cost supportive screen, but it should not be used as a stand-alone definition of hypervirulence (Russo et al., 2024; Wahl et al., 2024). A second-tier approach should add targeted polymerase chain reaction (PCR) or multiplex detection of *iucA*, *iroB*, peg-344, *rmpA*, and *rmpA2* for high-risk isolates, while WGS, capsule-locus typing, and multilocus sequence typing (MLST)/core-genome MLST (cgMLST) should be reserved for reference-laboratory confirmation, unusual resistance–virulence combinations, or outbreak investigation (Russo et al., 2024; Tang et al., 2025).

## 4 Therapeutic challenges and emerging options

Therapeutic management of CR-hvKp is difficult because the clinician must treat two linked problems: restricted antimicrobial susceptibility and a phenotype associated with deep-seated, metastatic, or rapidly progressive infection. Current CRE guidance is mechanism-based; carbapenemase identification is central because preferred agents differ for KPC-, OXA-48- like-, and metallo-β-lactamase-producing isolates (Tamma et al., 2024). For KPC-producing *K. pneumoniae*, ceftazidime-avibactam, meropenem-vaborbactam, and imipenem-cilastatin-relebactam are preferred options, reflecting improved outcomes and lower toxicity compared with older polymyxin- or aminoglycoside-based approaches (Tamma et al., 2024). Nevertheless, this evidence should be applied cautiously to CR-hvKp. Most treatment data come from CRE or CRKP cohorts rather than prospectively defined CR-hvKp populations, and meta-analytic evidence favoring ceftazidime-avibactam over polymyxins is largely observational and not pathotype-specific (Yang et al., 2023; Zhuang et al., 2023, 2025). Virulence status should therefore increase clinical vigilance, prompt assessment for deep or metastatic foci, and support early source control, but antimicrobial choice should remain anchored to carbapenemase class, susceptibility profile, infection site, and patient severity.

The therapeutic gap is wider for isolates producing NDM, VIM, or IMP enzymes because avibactam, relebactam, and vaborbactam do not inhibit metallo-β-lactamases. Infectious Diseases Society of America (IDSA) guidance supports ceftazidime-avibactam plus aztreonam or cefiderocol for metallo-β-lactamase (MBL)-producing Enterobacterales infections when susceptibility and clinical context are appropriate (Tamma et al., 2024). Cefiderocol showed activity in patients with serious carbapenem-resistant Gram-negative infections in the CREDIBLE-CR trial, but clinical interpretation remains cautious because outcomes varied by pathogen group and infection severity (Bassetti et al., 2021). Aztreonam-avibactam is an important emerging option because aztreonam resists MBL hydrolysis while avibactam protects it from many co-produced serine β-lactamases (Carmeli et al., 2025; Daikos et al., 2025).

Resistance emergence during therapy is an important management concern, but its true frequency in CR-hvKp is difficult to estimate because most evidence comes from case reports, in-host evolution studies, and broader CRE experience rather than prospective CR-hvKp cohorts. Mechanisms that may compromise newer β-lactam/β-lactamase inhibitor activity include KPC variants with altered inhibitor susceptibility, acquisition or co-production of metallo-β-lactamases, additional serine β-lactamases, and permeability changes affecting outer-membrane porins (Shi et al., 2024; Tamma et al., 2024). The report of KPC-135-mediated ceftazidime-avibactam resistance in ST11-K47 hypervirulent *K. pneumoniae* illustrates that resistance can arise within convergent resistance–virulence backgrounds rather than only in classical CRKP lineages (Shi et al., 2024). In-host evolution during tigecycline and polymyxin exposure has also been documented in CR-hvKp, and colistin resistance may emerge under treatment pressure in CR-hvKp (Jin et al., 2021; Zhang et al., 2021). Clinically, these observations support repeat antimicrobial susceptibility testing, reassessment of the carbapenemase mechanism when response is poor, avoidance of functionally weak monotherapy for high-inoculum or poorly controlled infection, and early source control whenever feasible.

For severe CR-hvKp disease, antimicrobial therapy should be paired with early source control, drainage of deep collections, assessment for metastatic foci, and repeat susceptibility or molecular testing when clinical response is poor (Russo and Marr, 2019; Choby et al., 2020; Tamma et al., 2024). Emerging agents do not replace rapid diagnostics, stewardship, or infection-site-specific judgment.

## 5 Current gaps and future directions

A major unresolved gap is the absence of a universally accepted operational definition for CR-hvKp. Recent work supports the combined detection of *iucA*, *iroB*, *peg-344*, *rmpA*, and *rmpA2* as the most pragmatic genomic approach for identifying hvKp, but the same studies emphasize that this marker set does not fully capture clinical virulence across all lineages and settings (Russo et al., 2024; Tang et al., 2025). Future definitions should integrate genotype, phenotype, infection syndrome, and outcome data rather than treating hypervirulence as a binary laboratory label.

Surveillance also remains uneven. Global genomic analysis has shown that canonical CR-hvKp isolates are already distributed across multiple countries and that plasmid-lineage combinations, including IncFIIK34 KPC-2 plasmids, may contribute to international dissemination (Jiang et al., 2025b). Yet many laboratories still report carbapenem resistance without parallel virulence profiling, which can delay recognition of convergent strains during outbreaks or severe invasive infections (Wahl et al., 2024). A practical future direction is the adoption of tiered surveillance in which routine antimicrobial susceptibility testing (AST) and carbapenemase testing are linked to targeted virulence PCR and referral sequencing for high-risk isolates.

However, the current evidence base remains geographically uneven. Several influential CR-hvKp reports derive from Asian healthcare settings, including blaKPC-2 acquisition by hvKp in Singapore and the expansion of ST11/KPC-producing CRKP lineages with virulence features in China (Chen et al., 2020; Zhang et al., 2020; Zhou et al., 2020). These observations are biologically important but should not be treated as a universal epidemiologic template. In the EU/EEA, recent public-health concern has centered on hvKp ST23-K1 carrying carbapenemase genes, with reporting countries increasing from four to ten and submitted isolates from 12 to 143 in the 2024 ECDC update (European Centre for Disease Prevention and Control, 2024). In the United States, a multicentre genomic analysis of 884 CRKP isolates identified only six virulence-plasmid–harboring CRKP isolates across ST23, ST893, and ST11 (Jiang et al., 2025a). Therefore, diagnostic thresholds and therapeutic inferences should be applied as local risk-stratification tools rather than globally generalizable algorithms.

Another gap is the limited resolution with which plasmid transmission is monitored in routine epidemiology. Mobilizable plasmids can carry a large proportion of acquired virulence genes in *K. pneumoniae*, suggesting that surveillance focused only on self-transmissible resistance plasmids may underestimate the mobility of virulence cargo (Zhang et al., 2023). Long-read sequencing, plasmid reconstruction, and standardized reporting of resistance–virulence plasmid structures should therefore become part of reference-laboratory investigation for CR-hvKp clusters.

Therapeutic evidence is another weak point. Current CRE guidance is organized mainly around carbapenemase class and susceptibility profile, but it does not provide CR-hvKp-specific treatment algorithms because prospective clinical data for this pathotype remain scarce (Tamma et al., 2024). Future studies should evaluate whether hypervirulence markers predict metastatic complications, relapse, mortality, or the need for longer treatment and more aggressive source control (Wang et al., 2024; Xu et al., 2025; Zheng et al., 2025; Zhu et al., 2026).

Prevention and non-traditional therapies should be framed as complementary research priorities rather than immediate substitutes for optimized antimicrobial therapy and source control. Phage therapy and phage-derived capsule depolymerases are attractive because they can provide strain- or capsule-directed activity, but their clinical translation is limited by narrow host range, bacterial escape, phage selection, dosing, manufacturing, and regulatory challenges (Kou et al., 2024). Monoclonal antibodies targeting capsular or surface polysaccharides provide useful preclinical proof-of-concept for antibody-mediated protection against carbapenem-resistant *K. pneumoniae*, but their clinical role in heterogeneous CR-hvKp populations remains undefined (Diago-Navarro et al., 2018; Opoku-Temeng et al., 2019). Vaccines are also promising for prevention, especially in high-risk populations, yet capsule and O-antigen diversity, geographic variation in antigen distribution, and the lack of late-stage efficacy data remain important barriers (Opoku-Temeng et al., 2019; Douradinha, 2024). The immediate priority is therefore an integrated framework linking rapid diagnostics, genomic surveillance, antimicrobial stewardship, infection prevention, and clinically anchored outcome studies (Li et al., 2022; Liu et al., 2023; Sheng et al., 2024).

## 6 Conclusion

Carbapenem-resistant hypervirulent *Klebsiella pneumoniae* represents a high-risk convergence of AMR, invasive virulence, and bacterial adaptability. Rather than emerging through one fixed pathway, it reflects repeated interactions among carbapenemase-encoding elements, virulence plasmids, high-risk clonal backgrounds, capsule variation, mobile genetic elements, and host or hospital selection pressures. For clinical microbiology, the key practical challenge is that no single feature reliably defines this pathotype. Carbapenem resistance establishes therapeutic risk, whereas hypermucoviscosity, capsule type, sequence type, and individual virulence genes are informative but incomplete markers. A defensible approach integrates antimicrobial susceptibility testing, carbapenemase classification, virulence-marker profiling, capsule and sequence typing, and WGS when invasive disease, treatment failure, unusual resistance–virulence combinations, or suspected transmission is present. Treatment should remain mechanism-based and clinically contextual, guided by carbapenemase class, susceptibility profile, infection site, source control, patient severity, and risk of resistance emergence. Because current evidence remains geographically uneven, regional validation of diagnostic markers, lineage-risk assumptions, and treatment outcomes is essential before CR-hvKp recommendations can be considered globally generalizable. The major remaining needs are standardized definitions, resource-adapted virulence-aware surveillance, plasmid-resolved outbreak investigation, and prospective outcome-focused treatment data. Progress will depend on linking rapid diagnostics, genomic epidemiology, antimicrobial stewardship, infection prevention, and clinical outcomes into a coordinated framework for earlier recognition and safer management.
